# Multidrug resistant superbugs in pyogenic infections: a study from Western Rajasthan, India

**DOI:** 10.11604/pamj.2021.38.409.25640

**Published:** 2021-04-29

**Authors:** Jitu Mani Kalita, Vijaya Lakshmi Nag, Sarika Kombade, Kavita Yedale

**Affiliations:** 1 Department of Microbiology, All India Institute of Medical Sciences, Jodhpur, Rajasthan, India

**Keywords:** Pyogenic infection, surgical site infection, superbug, multidrug resistance

## Abstract

**Introduction:**

the crude mortality rate due to infectious diseases in India is approximately 417 per one lakh persons and pyogenic infections are one of the significant contributor. Poor antimicrobial stewardship in India has led to an increase in multidrug resistant superbugs in both community as well as hospital settings. The aim of this study was to identify the bacterial etiology of pyogenic infections and to evaluate their antimicrobial resistance profile.

**Methods:**

this is a retrospective observational study from January, 2018 to December, 2018. A total 1851 samples, collected as a part of patient care were included in this study. Specimens were subjected to culture on Blood agar and MacConkey agar and incubated at 37°C for 48 hours. Species identification was done as per standard laboratory protocol. Antimicrobial susceptibility testing was performed using Kirby-Bauer disc diffusion according to Clinical and Laboratory Standards Institute guidelines.

**Results:**

of total 1851 samples, culture was positive in 61.54%. A total 70.59%, Gram negative organisms were isolated followed by Gram positive cocci in 45.48%, yeast in 1.05%, coryneform bacteria in 0.79% and in one case, non-tubercular mycobacteria was isolated. Staphylococcus aureus (30.9%) was the predominant organism isolated. Most common multi drug resistant isolates were Klebsiella spp. (74.79%) and Acinetobacter spp. (74.32%).

**Conclusion:**

this study gives an insight about the prevalence and common etiology of pyogenic infections along with their antimicrobial resistance profile in north western region of India. This study will contribute in formulating antibiotic stewardship program by selecting the antibiograms of pyogenic isolates.

## Introduction

Pyogenic infections are characterized by local inflammation of skin, soft tissue and bodily parts which are mainly caused by invasion and multiplication of pathogenic microorganism. These pathogen releases certain cellular or toxic metabolites and leukocidins which destroy neutrophils forming abscess and pus. Impetigo, osteomyelitis, septic arthritis, spondylodiscitis, otitis media, cystitis, meningitis, surgical site infections are common pyogenic infections. *Staphylococcus aureus, Streptococcus pyogenes, Escherichia coli, Klebsiella spp. Proteus spp*. and *Pseudomonas spp*. are the common etiological agents implicated in pyogenic infections [1]. Pyogenic infections are not only the leading cause of morbidity and mortality but also responsible for prolonged hospital stay and disability worldwide [2].

The crude mortality rate due to infectious diseases in India is approximately 417 per one lakh persons [3]. Over the years, the poor antimicrobial stewardship in India has led to an increase in multidrug resistant (MDR) superbugs in both community as well as hospital settings [4]. Pyogenic infections including surgical site infections are significant subgroup of infections encountered by infectious disease physicians in the hospitals worldwide. Although the diagnostic techniques are advanced now a days but treatment of pyogenic infections is challenging due to the emergence of MDR superbugs especially in developing countries. Most importantly, methicillin resistant *Staphylococcus aureus* along with the MDR Gram negative isolates are observed to be increasingly associated with pyogenic infections in recent years [5]. The appropriate knowledge of the pathogens, their antimicrobial resistance character and their updated antimicrobial therapy plays a crucial role in the treatment process as well as in infection control measures [6]. Therefore, this study was intended to identify the bacterial etiology of pyogenic infections and to ascertain the current scenario of antimicrobial resistance in these isolates in order to optimize empirical therapy.

## Methods

This is a retrospective observational study conducted in the Department of Microbiology at a tertiary care hospital in Jodhpur city from north western region of India from January, 2018 to December, 2018. A total of 1851 samples (pus aspirate and wound swab) were received which were collected as a part of patient care from various outpatient departments, inpatient departments and intensive care units for aerobic culture in Bacteriology laboratory during the study period. Specimens were subjected to culture on Blood agar and MacConkey agar media and incubated at 37°C for a period of 48 hours. Species identification was done on the basis of colony characteristics and various biochemical tests as per standard laboratory protocol.

In some cases Automated Siemens Microscan Walkaway (Beckman Coulter version 2.1) identification system was used. Antibiotic susceptibility testing was performed using Kirby-Bauer disc diffusion method as recommended by the Clinical and Laboratory Standards Institute (CLSI). Gram negative isolates were tested against aminoglycosides, cephalosporins, quinolones, piperacillin/tazobactam, carbapenems and Cotrimoxazole and Gram positive isolates were tested against penicillin, aminoglycosides, quinolones, erythromycin, clindamycin, and cotrimoxazole. Minimum inhibitory concentration (MIC) of colistin and vancomycin was detected by using E-strip method (Hi-Media Laboratories, Mumbai). Methicillin resistance among *Staphylococcus aureus* isolates was tested by using cefoxitin disc (30µg). Interpretation was done according to CLSI guideline, 2017-18. This study was approved by hospital ethics committee.

**Statistical analysis:** the interpretation and analysis of the data were done by using Microsoft Excel. The quantitative data were expressed as numbers and percentages in tabular form and figures.

## Results

**Figure 1:**
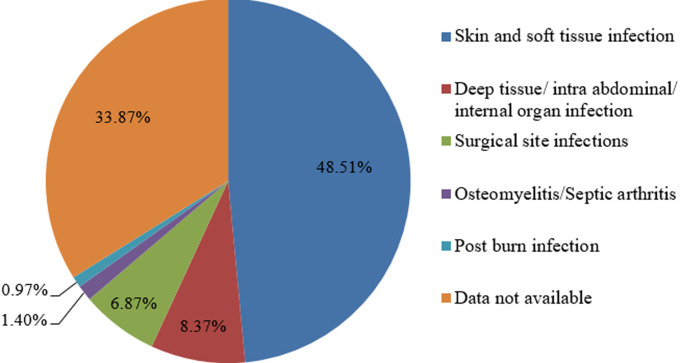
distribution of infection type

Out of total 1851 samples received, 1137 (61.43%) samples were from male patient. Samples were received from patients of all age with most commonly from 21-40 years of age group (34.31%). Out of total samples received, 1398 (75.53%) were wound swab and 453 (24.47%) were pus aspirate from various infections. Maximum number of samples was received from patients with skin and soft tissue infection (48.51%) and data was not available in 33.87% samples. Distribution of various infection types are shown in Figure 1.

Culture was positive in 1139 (61.54%) samples, mixture was reported in 57 (3.08%), contaminant in 43 (2.32%), and sterile in 612 (33.06%) samples. Mixture was reported when there was growth of more than two organisms. Among total culture positives, total 804 (70.59%), Gram negative organisms were isolated followed by Gram positive cocci 518 (45.48%), yeast 12 (1.05%), coryneform bacteria 9 (0.79%) and in one case non tubercular mycobacteria was isolated. *Staphylococcus aureus* 352 (30.9%) was the predominant isolate followed by *Escherichia coli* 282 (24.76%). Distribution of organism is shown in Table 1. Most of the Gram negative isolates showed high resistance towards cephalosporin, cotrimoxazole and quinolones and Gram positive cocci showed high resistance towards penicillin and quinolone group of drugs. Multidrug resistance (MDR) was observed in most of the commonly isolated organism.

**Table 1:** distribution of microorganisms

Group	Species	Total number n (%)
Gram negative fermenter (Enterobacteriaceae) n=511	*Escherichia coli*	282 (24.76)
	*Klebsiella spp*.	164 (14.4)
	*Enterobacter spp*.	20 (1.76)
	*Proteus spp*.	18 (1.58)
	*Providencia spp*.	5 (0.45)
	*Morganellamorganii*	3 (0.26)
	*Citrobacter spp*.	17 (1.49)
	*Serratiamarcescens*	1 (0.08)
	*Tatumella spp*.	1 (0.08)
Gram negative non-fermenter n=293	*Pseudomonas spp*.	190 (16.68)
	*Acinetobacter spp*.	95 (8.34)
	*Burkholderiacepacia complex*	3 (0.26)
	*Ralstoniapicketti*	4 (0.35)
	*Sphingomonas spp*.	1 (0.08)
Gram positive cocci n=518	*Staphylococcus aureus*	352 (30.9)
	Coagulase negative *Staphylococci*	103 (9.04)
	Group A *Streptococcus*	6 (0.53)
	*Enterococcus spp*.	53 (4.65)
	Other Beta hemolytic *Streptococci*	4 (0.35)
Coryneform bacteria n=9	*Corynebacterium spp*.	9 (0.79)
Yeast n=12	*Candida spp*.	12 (1.05)
NTM n=1	*Mycobacterium fortuitum*	1 (0.08)

**Figure 2:**
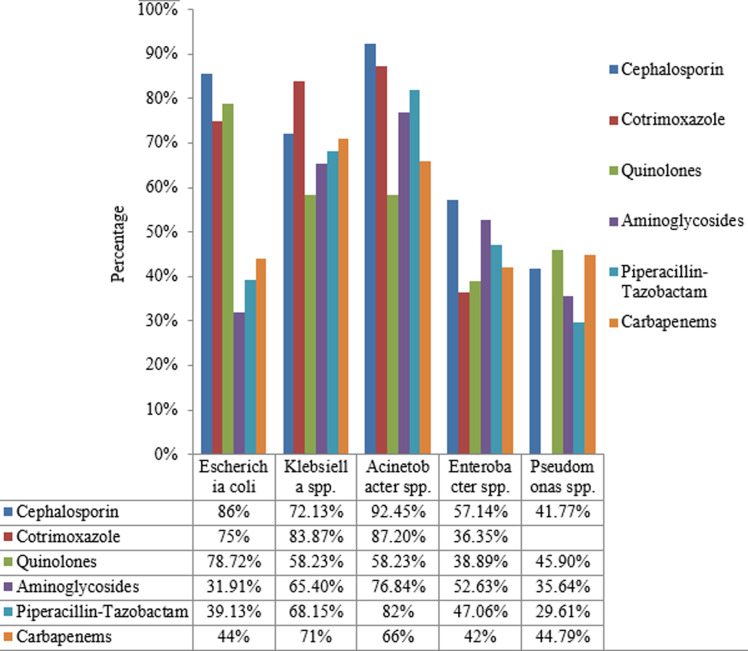
resistance pattern among Gram negative organisms (Cotrimoxazole is intrinsically resistant to *Pseudomonas spp*.)

**Figure 3:**
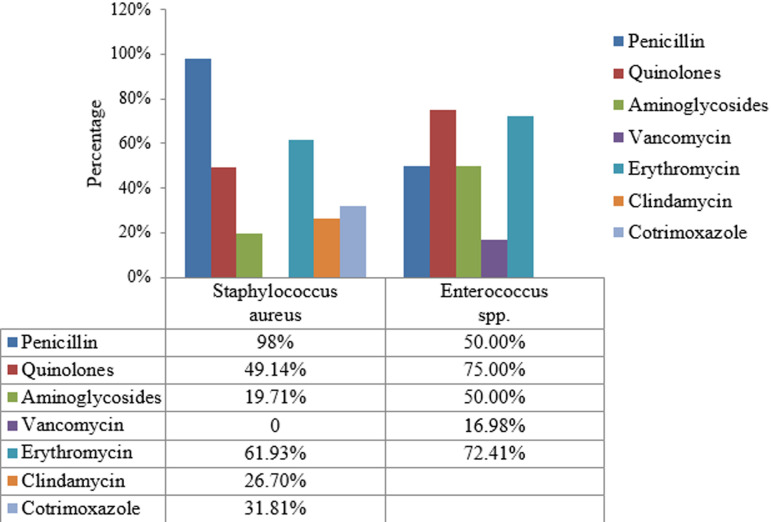
resistance pattern among Gram positive organisms (Clindamycin and Cotrimoxazole are not recommended for *Enterococcus spp*.)

**Figure 4:**
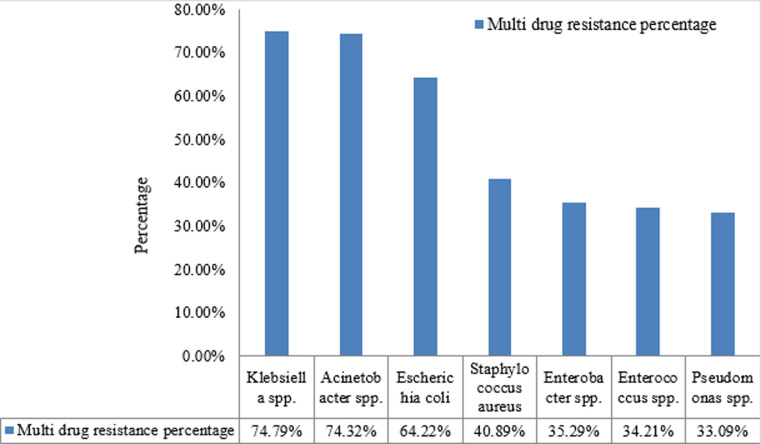
distribution of multi drug resistance among commonly isolated bacteria

None of the tested Gram negative isolate showed resistance towards colistin except for the intrinsically resistant ones. Among *Staphylococcus aureus* isolates, 151 (13.26%) isolates were methicillin resistant and inducible clindamycin resistance was observed in 57 (16.19%) isolates. Among *Enterococcus spp*.16.98% were vancomycin resistant. Antimicrobial susceptibility pattern of most commonly isolated Gram positive and Gram negative isolates is shown in Figure 2 and Figure 3. Most common MDR isolates were *Klebsiella spp*. (74.79%) and *Acinetobacter spp*. (74.32%) in this study. MDR bacterial isolates were identified according to the criteria recommended by international expert committee of the European Centre for Disease Prevention and Control and the Centers for Disease Control and Prevention [7]. Isolates resistant to at least one antimicrobial from three different groups of drugs tested was considered as MDR. Distribution of common MDR isolates is shown in Figure 4.

## Discussion

Pyogenic infections including surgical site infections represent a significant burden in terms of cost to health services around the world. Because of irrational use of antimicrobial agents, infections due to multi drug resistant (MDR) bacterial isolates are becoming the biggest threat in the world, as there is very few newer antibiotic options are available. Although the evolution of resistant strains is a natural phenomenon, overuse and misuse of antimicrobial agents accelerate the emergence of MDR strains. The overall pyogenic wound infections in terms of significant bacterial growth in aerobic culture of clinical specimens among study subjects were 61.54%. Almost similar rate of culture positivity is also shown by other Indian study [8].

A study from Nepal also showed almost similar rate of culture positivity [6]. In this study highest numbers of samples were received from the patients of age group more than 50 years followed by 21-30 years. Male and female ratio is 1.6: 1. More numbers of patients (34.31%) were from 21-40 years of age group. Some other studies also showed similar findings [9, 10]. Although Gram negative organisms were more prevalent (70.59%) in this study but Gram positive cocci, *Staphylococcus aureus* (30.9%) was the most common organism isolated of which 13.26% were Methicillin resistant *Staphylococcus aureus* (MRSA). Some other studies also showed similar findings [6, 9-11].

A study by Indian council of medical research antimicrobial resistance surveillance network (ICMR-AMRSN) also reported *S. aureus* to be the most frequently isolated organism from patients with skin and soft tissue infections (73.7%) but much higher than the present study [12]. In hospital, the sources of *S. aureus* may be from the high carriage rate of *S. aureus* in patient and healthcare worker, hands of patients and healthcare worker or inanimate objects. Again, due to presence of *S. aureus* as normal flora of human body, there is possibility of endogenous infections as well. The MRSA prevalence in the present study was much lower than the recently reported prevalence of 37.3% by ICMR AMRSN and 40% as reported by Indian Network for Surveillance of Antimicrobial Resistance (INSAR) group study [12, 13]. However these prevalence of MRSA were reported from various samples. *Enterococcus spp*. (4.65%) was second most common Gram positive organism frequently isolated in this study. Among Gram negative isolates, *Escherichia coli* (24.76%) was the most prevalent organism followed by *Pseudomonas spp*. (16.68%), *Klebsiella spp*. (14.4%) and *Acinetobacter spp*. (8.34%) respectively.

A study from south India also showed *E. coli* to be the most prevalent organism among Gram negative isolates [14]. However a study from western India showed *Pseudomonas spp*. to be the most commonly isolated Gram negative bacteria [9]. It is well known that *S. aureus* and Gram negative bacterial pathogens can produce very potent virulence factors, responsible for maintaining the infection and delaying the process of wound healing [15]. Therefore, present study results confirm the usual most prevalent microorganisms isolated in pyogenic infections. Gram negative bacteria have also been described to be commonly associated with various nosocomial infections including surgical site infection. High rates of antimicrobial resistance among the pathogenic bacteria associated with the pyogenic infections are major concerns of this study. India was the highest antibiotic consumer, with 10.7 units being consumed per person in 2010. India registered an increase of 23% in the retail sale volume of antibiotics among BRICS (Brazil, Russia, India, China and South America) countries [16]. Therefore, over the counter availability and inappropriate antibiotic abuse has resulted in the development of antimicrobial resistance as well as MDR superbugs, which is difficult to treat.

Most of the Gram negative isolates were resistant to many of the antibiotics tested. Among Gram positive organisms MRSA isolates showed maximum resistance pattern. None of the MRSA isolate showed resistance towards vancomycin. Vancomycin resistant Enterococcus (VRE) was isolated in 16.98% cases. Some other Indian studies showed much lower rate of isolation of VRE than present study [17, 18]. Among Gram negative isolates, overall cephalosporin, cotrimoxazole and quinolones were the most resistant group of antibiotics and in Gram positive isolates, penicillin and quinolones were the most resistant group. None of the tested Gram negative isolate showed resistance towards colistin except those having intrinsic resistance. Cephalosporin and quinolone group of antibiotics showed high resistance may be because these were the most commonly prescribed antimicrobials in local hospital settings.

A recent Indian study on nationwide surveillance of antimicrobial resistance of common Gram negative bacteria isolated from various samples showed >70% resistance towards cephalosporin and >60% resistance towards commonly used quinolones along with high resistance towards carbapenems specially in *Klebsiella pneumoniae* and *Acineobacter baumanii* [19]. In this study as well, MDR was observed more in Gram negative superbugs like *Klebsiella spp*. (74.79%) and *Acinetobacter spp*. (74.32%). Findings of this study indicate the existence of high MDR superbugs in pyogenic wound infections. As this is a tertiary care hospital, patient might exposed to common antibiotics before admission and inappropriate infection control procedures in the hospitals might be the cause of rising rates of resistance among these bacteria.

Limitations of this study are firstly, this is a retrospective study in which we couldn´t look into the significant determinants such as the source of infection, the duration of hospital stay, and clinical outcome. Secondly this study was based on characterization of bacterial isolates based on phenotypic conventional and automated methods only. Molecular characterization of MDR bacterial isolates would have generated more useful epidemiological results.

## Conclusion

This study gives an insight about the prevalence and common etiology of pyogenic infections along with their antimicrobial resistance profile in the north western region of India. In this study *S. aureus, E. coli, Klebsiella spp. Pseudomonas spp*. and *Acinetobacter spp*. were most commonly isolated bacteria from pyogenic wound infections. Although *S. aureus* was the most predominant bacteria isolated but most of the multi-drug resistant (MDR) superbugs were Gram negative organism in this study. Continuous surveillance is necessary to update the knowledge of resistance profiles of clinical isolates to provide the most appropriate dose regimen and to make local antibiotic policy against pyogenic infections so that MDR bacterial infections can be managed early. This study will contribute in formulating antibiotic stewardship programme of this hospital by selecting the antibiograms of pyogenic isolates and restricting the armament of last reserve antimicrobials.

### What is known about this topic

- Pyogenic infections are one of the major health hazards worldwide;
- Surgical site infections are important health care associated infections and contributed in significant morbidity and mortality;
- Staphylococcus aureusis the most common organism implicated in pyogenic infections including surgical site infections.

### What this study adds

- This study highlights emergence of multidrug resistant Gram negative superbugs from pyogenic infections in north western region of India;
- Most of the Gram negative superbugs isolated from pyogenic infections are resistant to commonly used empirical antimicrobials in this study;
- This study shows isolation of some uncommon organisms from pyogenic infections which are mainly related to health care associated infections. Strict infection control practices should be implemented to prevent these infections.
